# Metabolic syndrome in a Taiwanese metropolitan adult population

**DOI:** 10.1186/1471-2458-7-239

**Published:** 2007-09-13

**Authors:** Cheng-Chieh Lin, Chiu-Shong Liu, Ming-May Lai, Chia-Ing Li, Ching-Chu Chen, Pei-Chia Chang, Wen-Yuan Lin, Yih-Dar Lee, Tsann Lin, Tsai-Chung Li

**Affiliations:** 1 Department of Family Medicine, China Medical University Hospital, Taichung, Taiwan; 2 Institute of Health Care Administration, College of Health Science, Asia University, Taichung, Taiwan; 3 Medical Research, China Medical University Hospital, Taichung, Taiwan; 4 Division of Endocrinology and Metabolism, Department of Medicine, China Medical University Hospital, Taichung, Taiwan; 5 Lilly Taiwan, Eli Lilly and Company, Taipei, Taiwan; 6 Department of Family Medicine, College of Medicine, China Medical University, Taichung, Taiwan; 7 Institute of Chinese Medicine Science, China Medical University, Taichung, Taiwan; 8 Biostatistics Center, China Medical University, Taichung, Taiwan

## Abstract

**Background:**

Metabolic syndrome (MS) is a combination of medical disorders that increase one's risk for cardiovascular disease and diabetes. Little information exists on the prevalence of MS in a general adult population in Taiwan.

**Methods:**

We did a cross-sectional survey in a representative sample of 2,359 Chinese adults aged 40 years and over who lived in a metropolitan city, Taiwan in 2004–05. MS was defined by Adult Treatment Panel III criteria modified for Asians.

**Results:**

The prevalence of MetS was 35.32% and 43.23% in men aged 40–64 years and 65 years and over, respectively, and 24.19% and 51.82% in women aged 40–64 years and 65 years and over. Older age, postmenopausal status, higher body mass index, current smoking, low education attainment, low household income, no alcohol consumption, lower level of occupation physical activity, and a family history of diabetes were associated with increased odds of MetS.

**Conclusion:**

MetS was present in more than 30% of the Taiwan adult population aged 40 years and over in a metropolitan area; there were substantial variations by age and body mass index groups.

## Background

Cerebrovascular disease (CVA), cardiovascular heart disease (CHD), and diabetes are the most prominent causes of death worldwide. In Taiwan, CVA, CHD, and diabetes were the number 2, 3 and 4 leading causes of death in men and women in 2005, accounting for more than 25% and 40% of total deaths, respectively [1].

Factors associated with an increased risk of developing CHD that tend to cluster in individuals include abdominal obesity, high blood pressure, a low level of high-density lipoprotein (HDL) cholesterol, a high triglyceride level, and a high plasma glucose concentration [2-4]. These associated risk factors have been called the metabolic syndrome (MetS). Insulin resistance due to obesity or an inherited genetic defect has been hypothesized as the mechanism underlying MetS. With changes in lifestyle and diet in Taiwan, the prevalence of obesity has increased dramatically. In consequence, the prevalence of MetS may be expected to increase markedly.

Estimates of the prevalence of MetS have varied substantially in part because of the variability of populations in a geographic region, urbanization, and diagnostic criteria. We aimed to estimate the prevalence of MetS in a representative sample of a Taiwanese metropolitan general adult population and to examine distributions of MetS by age, body mass index (BMI), and socioeconomic and lifestyle factors.

## Methods

### Population and participants

This was a population-based cross-sectional study. The target population consisted of residents aged 40 and over in Taichung City, Taiwan, who were investigated in October, 2004. Taichung is a city located in west-central Taiwan with a population of just over one million people, making it the third largest city on the island. The area of Taichung city is 163.4256 km^2^, and its population density was 6249/km^2 ^in 2004. There were a total of 363,543 residents aged 40 and over in this area during the time of the study, about 4.09% of the national population of the same age. The sampling frame of this study was the set of all individuals' records from the Bureau of Households. A two-stage sampling design was used to draw residents, with a sampling rate proportional to size (SRPS) within each stage. At each stage, simple random sampling was used. In the first stage of sampling, the sampling unit was Li (administrative units, equivalent to blocks of household units) and the selection probability for Li was set at 0.125. Lis were randomly selected from each city district (a total of 8 city districts), yielding a total of 39 Lis selected.

In the second stage, 110 individuals were randomly selected from each sample Li. A total of 4280 individuals were selected. During household visits, we identified 750 individuals who were not eligible and excluded them from study sample. The reasons for exclusion included death (n = 18), hospitalization or imprisonment (n = 14), living abroad (n = 39), moving out of the area (n = 411), living in their children's home (n = 7), sampling frame mistakes (n = 59), and not being at home during 3 visits made by interviewers (n = 202). A total of 3,530 subjects were eligible, and 2,359 agreed to participate and provide complete information. Thus the overall response rate was 66.83%. This study was approved by the Human Research Committee of China Medical University Hospital. Written informed consent was obtained from each participant.

### Anthropometric measurement and laboratory examination

Anthropometric measurements and blood samples were obtained from the complete physical examination. Weight and height were measured on an autoanthropometer (super-view, HW-666), with the subjects shoeless and wearing light clothing. Body mass index (BMI) was derived from the formula of weight (kg) ÷ (height)^2 ^(m^2^). With the participant standing, waist circumference was measured midway between the iliac and the costal margin, and hip circumference at its maximum, and then the waist-to-hip ratio was calculated. Blood pressure was measured using an electronic device (COLIN, VP-1000, Japan).

Blood was drawn with minimal trauma from an antecubital vein in the morning, after a 12-hour overnight fasting, and was sent for analysis within four hours of collection. Biochemical markers such as HDL-cholesterol, triglyceride, fasting glucose, fasting insulin, urine albumin and creatinine were analyzed by a biochemical autoanalyzer (Beckman Coluter, Lx-20, USA) at the Clinical Laboratory Department of China Medical University Hospital.

### Variables definition

Data on sociodemographic characteristics, including gender, age, educational attainment, marital status, household income, smoking, drinking, physical activity, occupational activity, menopausal status, dietary habits, family history of cardiovascular-related diseases, physician-diagnosed diseases, and medication history were collected when the participants underwent a complete physical exam. Educational level was divided into 3 categories: less than 9 years, 9 to 12 years, and more than 12 years. Marital status was divided into 2 categories: currently married and currently unmarried (including single, widowed, divorced or separated). Economic status was divided into 3 categories according to the participant's monthly household income: NT40000 or less, NT40000 to 100000, and more than NT100000. Questions on physical activity were separated into two sections: recreational and occupational/household activity in the previous year.

Smoking was categorized as current, past, and never. Past smokers were those who had smoked at least 100 cigarettes during their lifetime but who did not currently smoke cigarettes. Drinking was categorized as current, never, and past. Never drinkers were those who self-reported they did not regularly drink beer, wine, or hard liquor.

### The metabolic syndrome

A modification of the Third Report of the National Cholesterol Education Program's Adult Treatment Panel definition of MetS was used [4]. The ATP III defined MetS as the presence of three or more of the following: fasting plasma glucose ≥ 110 mg/dl, serum triglycerides ≥ 150 mg/dl, serum HDL-cholesterol <40 mg/dl in men and <50 mg/dl in women, blood pressure ≥ 130/85 mmHg, or waist circumference >90 cm in men and >80 cm in women.

### Statistical analysis

Continuous variables were reported as mean ± standard deviation (SD) and categorical variables were reported as percentage (95% confidence intervals, abbreviated as CI). Differences in proportions and means were assessed using a χ^2 ^test or t test. Prevalence was expressed with 95% CI. Weighted prevalence according to proportionate sampling of age and gender was calculated. All reported p values were those of two-sided tests; statistical significance was set at p < 0.05. All analyses were performed using SAS version 8.0 (SAS Institute Inc, Cary, NC).

## Results

The anthropometric characteristics and self-reported illnesses of the participants are summarized in Table 1, and the sociodemographic characteristics are in Table 2. The overall prevalence of MetS in the study sample was 37.66% for men and 29.21% for women, as defined by ATP III guidelines (p < 0.001). The prevalence of individual components of MetS and the number of metabolic abnormalities stratified by quartiles of BMI is shown for men and women in Table 2. The prevalence of MetS stratified by age is presented in Table 3. The prevalence of high TG levels, high BP and high fasting glucose levels was higher in those aged greater than or equal to 65 years than those aged 40–64 years in both sexes. The prevalence of abdominal obesity was significantly higher in the older group of women. The prevalence of MetS in men was 35.32% (95% CI: 32.02%, 38.62%) in those aged 40–64 years, and 43.23% (37.95%, 48.50%) for those aged 65 years and over; in women, the prevalence was 24.19% (21.53%, 26.85%) in those aged 40–64 years, and 51.82% (45.22%, 58.42%) for those aged 65 years and over. The weighted prevalence of MetS in men, women and overall was 36.85% (34.0%, 39.6%), 29.51% (27.02%, 32.01%), and 32.97% (31.09%, 34.85%).

**Table 1:** Anthropometric characteristics and self-reported illnesses in the study sample *

	Men (n = 1147)	Women (n = 1212)
Height (cm)	166.44 (166.09–166.80)	155.30 (154.99–155.60)
Weight (kg)	68.75 (68.15–69.34)	57.58 (57.09–58.07)
Body mass index (kg/m^2^)	24.77 (24.59–24.96)	23.88 (23.69–24.07)
Waist circumference (cm)	86.35 (85.85–86.86)	76.68 (76.18–77.18)
Systolic blood pressure (mmHg)	138.85 (137.65–140.05)	132.66 (131.38–133.94)
Diastolic blood pressure (mmHg)	82.57 (81.90–83.23)	75.49 (74.80–76.19)
Fasting blood glucose (mg/dl)	106.13 (104.41–107.84)	100.89 (99.37–102.41)
HDL-cholesterol (mg/dl)	41.49 (40.86–42.12)	50.21 (49.49–50.92)
Triglyceride (mg/dl)	136.61 (130.12–143.10)	107.31 (103.34–111.29)
Self-reported CHD (%)	15.30 (13.21–17.39)	12.83 (10.94–14.72)
Self-reported Stroke (%)	5.61 (4.27–6.95)	1.99 (1.20–2.78)
Self-reported Hypertension (%)	31.35 (28.66–34.04)	23.33 (20.95–25.71)
Self-reported Diabetes (%)	12.16 (10.27–14.05)	7.38 (5.90–8.86)
Family history of hypertension (%)	42.36 (39.49–45.23)	52.78 (49.96–55.60)
Family history of diabetes (%)	28.80 (26.16–31.44)	33.58 (30.91–36.25)
Menopausal status (%)		
Premenopausal	-	39.00 (36.24–41.76)
Postmenopausal	-	61.00 (58.24–63.76)

* Data are given mean (95% confidence interval), except where indicated otherwise.

**Table 2:** Sociodemographic characteristics of the study sample*

	Men	Women
Age (years)	58.56 (57.84–59.27)	55.24 (54.65–55.84)
Education (%)		
<9	28.87 (26.24–31.50)	44.24 (41.44–47.04)
9–12	47.42 (44.53–50.31)	42.50 (39.71–45.29)
>12	23.71 (21.24–26.18)	13.26 (11.35-15.17)
Marital status (%)		
Married	88.20 (86.33–90.07)	76.71 (74.32–79.10)
Not currently married	11.80 (9.93–13.67)	23.29 (20.90–25.68)
Household income, NT$/month		
40000	47.92 (45.01–50.83)	47.81 (44.94–50.68)
40000–100000	40.30 (37.44–43.16)	42.50 (39.66–45.34)
100001	11.78 (9.90–13.66)	9.68 (7.98–11.38)
Smoking (%)		
Never	48.25 (45.36–51.14)	95.29 (94.10–96.48)
Past	22.86 (20.43–25.29)	1.24 (0.62–1.86)
Current	28.88 (26.26–31.50)	3.47 (2.44–4.50)
Drinking (%)		
Never	54.10 (51.21–56.99)	88.52 (86.72–90.32)
Past	9.42 (7.73–11.11)	1.16 (0.56–1.76)
Current	36.47 (33.68–39.26)	10.32 (8.61–12.03)

* Data are given as percentage (95% confidence interval).

**Table 3:** The prevalence of the metabolic syndrome and its component abnormalities by age group in men and women

	Age					
		
	Men			Women		
				
	40–65 (n = 807)	≥ 65 (n = 340)	P-value	40–65 (n = 992)	≥ 65 (n = 220)	P-value
Large WC	28.5	32.06	0.2563	25.3	43.64	<0.0001
High Tg level	35.07	29.12	0.0595	19.25	33.18	<0.0001
Decreased HDL	52.79	52.94	1	56.35	62.73	0.0978
High BP	63.2	84.41	<0.0001	44.15	84.09	<0.0001
High glucose level	20.32	31.76	<0.0001	11.59	34.09	<0.0001
ATP III -Asia items (%)			0.0182			<0.0001
None	13.51	9.41		23.08	7.27	
One	26.39	20.29		31.35	17.73	
Two	24.78	27.06		21.37	23.18	
Three or more	35.32	43.23		24.19	51.82	

## Discussion

We used the ATP III criteria for MetS to evaluate its prevalence in an adult non-institutionalized civilian population of a metropolitan area in Taiwan. MetS was present in 33.32% of adults aged over 40 years. If the definitions of MetS recommended by ADA and IDF are adopted, the estimated prevalence would become 39.81% and 25.69%, respectively. Additionally, nearly 87.71% of men and 79.79% of women had one component of MetS by ATP III criteria. The prevalence of MetS was more common in women than in men among participants aged 65 and over (51.82% vs. 35.32%), but not among participants aged 40–65 years (24.19% vs. 35.32%). It was also higher in people with higher BMI among both men and women. These findings suggest that MetS is widespread among Taiwan adults aged 40 and over, and has become a serious public health challenge in Taiwan metropolitan areas.

A few representative surveys have been done in diverse Asian populations to estimate MetS [5-12]. In general, the prevalence found in studies in Asia is lower than in regions that are more economically developed [13-17], and the estimate of the prevalence in our study is closer to those in later studies. In a study using the same definition as ours, Chuang and colleagues identified a MetS prevalence of 23.77% in women and 17.73% in men, in a community-based survey of 8,320 men and women aged 30–92 years on an offshore island of Taiwan from 1991–1995 [11]. A possible explanation for the higher prevalence being observed in our study is that the much higher level of urbanization in the city we studied increased the prevalence of risk factors for CVA, such as obesity, decreased physical activity, smoking, hypertension, and diabetes. Hypertriglyceridemia, low HDL cholesterol, overall obesity, and smoking have been reported to be more prevalent in the urban population [18]. Gu's study also indicated that the prevalence of MetS and overweight was higher in urban than rural residents [6]. Another alternative explanation is that there exists an increasing trend in the prevalence of MetS due to the adoption of a modernized life style. As compared with a more recent study in 2002 [14], it reported a MetS prevalence of 13.6% in women and 18.3% in men from a national survey of 5,936 men and women aged 20–79.9 years. When we compared our estimates of age-specific prevalence with theirs, we still observed a slightly increasing trend, indicating the prevalence of this syndrome is still rising.

The age-adjusted prevalence of MetS in this Taiwanese population was higher in men (36.85%) than in women (29.51%), which was similar to previous findings [14]. In a previous report in a US population, there was little overall difference between men and women (24% versus 23.4%, respectively) [16].

The current study was performed with a representative sample of the Taiwanese adult population, using standard protocols and instruments. All participants underwent a complete physical check-up. To ensure the quality of the data collection, a strict personnel training process and vigorous quality assurance programs were set up. Therefore, the proportion of missing data due to lack of a blood specimen is pretty low (<0.5%), compared to that in previous survey in Taiwan (>10%) [12]. Additional strengths of the study include standard laboratory methods for the measurement of glucose and lipids, and the use of a central clinical laboratory for all glucose and lipid assays.

Two limitations of the study merit note. The principal limitation relevant to the interpretation of our results is the use of cross-sectional data; thus, causal pathways underlying the observed relationships cannot be inferred. Second, the response rate was 66.83%, indicating that potential selection bias might exist. To assess this possibility, we examined the demographic characteristics of the nonresponders; responders and nonresponders were compared by age, sex, and administrative unit, and similar distributions were found (age distribution for responders and nonresponders: 77.13% vs 74.55% for <65 years old and 22.87% vs 25.45% for <65 years old; sex distribution: 50.51% vs 48.86% for male and 49.49% vs 51.14% for female; most of the differences for distribution of administrative unit between sample and population is less than 1%, only 2 administrative units are higher: 3% and 6%). The non-differential distributions in age, sex, and administrative unit, indicate this kind of selection error might be random, thus, the biased results in the effect may be toward the null, a lesser threat to validity.

## Conclusion

In conclusion, metabolic syndrome was present in more than 30% of the Taiwanese adult population aged 40 years and over in a metropolitan area. The present study reveals the exceptionally high prevalence of the metabolic syndrome varied within age and gender groups. Comprehensive public health efforts are needed to reduce adverse levels of these risk factors in these high-risk subpopulations.

## Competing interests

The author(s) declare that they have no competing interests.

## Authors' contributions

CCL and TCL designed the study and drafted the manuscript. CSL, CIL, MML, CCC, WYL, and PCC carried out the study, participated in coordination and evaluation of data. YDL and TL contributed to the study with their knowledge on field study and helped to draft the manuscript. PCC, TCL, and CIL carried out the data organization and performed the statistical analysis. All authors read and approved the final manuscript.

## Pre-publication history

The pre-publication history for this paper can be accessed here:



## References

[B1] Department of Health, The Executive Yuan, ROC (2005). Statistics Report, Republic of China.

[B2] Chen J, Muntner P, Hamm LL, Jones DW, Batuman V, Fonseca V, Whelton PK, He J (2004). The metabolic syndrome and chronic kidney disease in U.S. adults. Ann Intern Med.

[B3] Isomaa B, Almgren P, Tuomi T, Forsén B, Lahti K, Nissén M, Taskinen MR, Groop L (2001). Cardiovascular morbidity and mortality associated with the metabolic syndrome. Diabetes Care.

[B4] (2001). Executive summary of the third report of the National Cholesterol Education Program (NCEP) expert panel on detection, evaluation, and treatment of high blood cholesterol in adults (Adult Treatment Panel III). JAMA.

[B5] Onat A, Ceyhan K, Başcar O, Erer B, Toprak S, Sansoy V (2002). Metabolic syndrome: major impact on coronary risk in a population with low cholesterol levels – a prospective and cross-sectional evaluation. Atherosclerosis.

[B6] Gu D, Reynolds K, Wu X, Chen J, Duan X, Reynolds RF, Whelton PK, He J, InterASIA Collaborative Group (2005). Prevalence of the metabolic syndrome and overweight among adults in China. Lancet.

[B7] Cameron AJ, Shaw JE, Zimmet PZ (2004). The metabolic syndrome: prevalence in worldwide populations. Endocrinol Metab Clin North Am.

[B8] Al-Lawati JA, Mohammed AJ, Al-Hinai HQ, Jousilahti P (2003). Prevalence of the metabolic syndrome among Omani adults. Diabetes Care.

[B9] Azizi F, Salehi P, Etemadi A, Zahedi-Asl S (2003). Prevalence of metabolic syndrome in an urban population: Tehran Lipid and Glucose Study. Diabetes Res Clin Pract.

[B10] Gupta A, Gupta R, Sarna M, Rastogi S, Gupta VP, Kothari K (2003). Prevalence of diabetes, impaired fasting glucose and insulin resistance syndrome in an urban Indian population. Diabetes Res Clin Pract.

[B11] Chuang SY, Chen CH, Tsai ST, Chou P (2002). Clinical identification of the metabolic syndrome in Kinmen. Acta Cardiol Sin.

[B12] Hwang LC, Bai CH, Chen CJ (2006). Prevalence of obesity and metabolic syndrome in Taiwan. J Formos Med Assoc.

[B13] Meigs JB, Wilson PW, Nathan DM, D'Agostino RB, Williams K, Haffner SM (2003). Prevalence and characteristics of the metabolic syndrome in the San Antonio Heart and Framingham Offspring Studies. Diabetes.

[B14] Park YW, Zhu S, Palaniappan L, Heshka S, Carnethon MR, Heymsfield SB (2003). The Metabolic Syndrome: Prevalence and associated risk factor findings in the US population from the third National Health and Nutrition Examination Survey, 1988–1994. Arch Intern Med.

[B15] Ford ES, Giles WH, Dietz WH (2002). Prevalence of the metabolic syndrome among US adults: findings from the third National Health and Nutrition Examination Survey. JAMA.

[B16] Laaksonen DE, Lakka HM, Niskanen LK, Kaplan GA, Salonen JT, Lakka TA (2002). Metabolic syndrome and development of diabetes mellitus: application and validation of recently suggested definitions of the metabolic syndrome in a prospective cohort study. Am J Epidemiol.

[B17] Lakka HM, Laaksonen DE, Lakka TA, Niskanen LK, Kumpusalo E, Tuomilehto J, Salonen JT (2002). The metabolic syndrome and total and cardiovascular disease mortality in middle-aged men. JAMA.

[B18] Abdul-Rahim HF, Husseini A, Bjertness E, Giacaman R, Gordon NH, Jervell J (2001). The metabolic syndrome in the West Bank Population. Diabetes Care.

